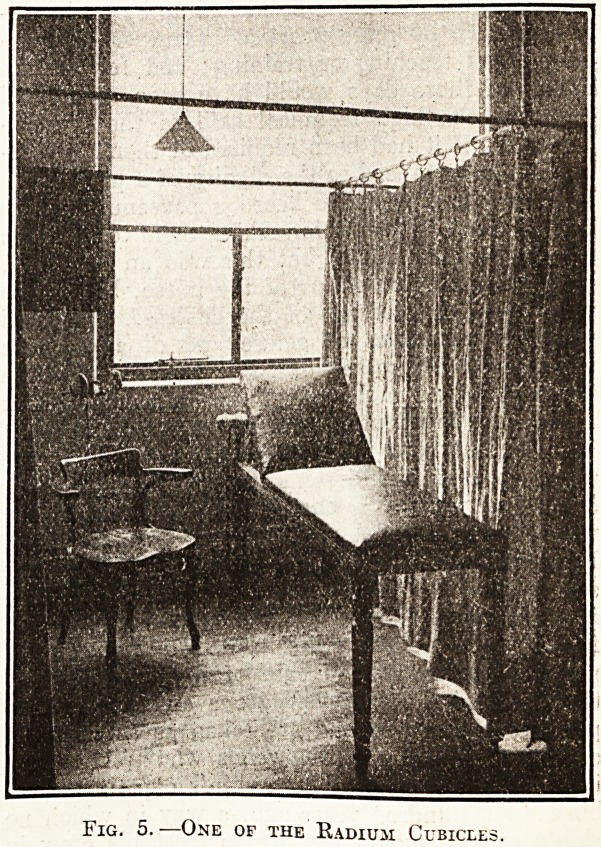# The Electrical and Radio-Therapeutic Department of the Cancer Hospital (Free), London

**Published:** 1914-08-01

**Authors:** Alfred M. Hooper

**Affiliations:** Assistant Secretary to the Hospital


### THE ELECTRICAL AND RADIO-THERAPEUTIC DEPARTMENT OF THE CANCER HOSPITAL (FREE), LONDON.

By ALFRED M. HOOPER, Assistant Secretary to the Hospital.

The first electrical department of this hospital was instituted in the year 1903, when an *x*-ray apparatus was installed and a commencement of its use made in the diagnosis and treatment of disease. For some years the work was carried on in two rooms in the main building, but the use of electrical methods and the introduction of radium in treatment increased to such an extent that it was decided to erect a special building for the department. The new premises, which were opened in 1912, occupy a site in the grounds apart from the hospital. The department is now housed in a red-brick building of two floors, but already it has been found inadequate and an additional floor is being added to accommodate wards for resident patients and further work-room for the physicist. Direct communication is being made with the hospital which will enable patients to be transferred to the department without entering the grounds.

### General Description.

On the ground floor are situated a large waiting-room, the radiographic room, director's consulting room, which contains a specially constructed safe built into the wall for the custody of the radium, physicist's room, and the photographic dark-room. On the first floor is a large treatment room, part of which is divided into cubicles by curtains; each cubicle is self-contained and has its own separate supply of gas, water, and electrical current, thus affording privacy for each case under treatment. There are also on this floor three separate *x*-ray cabinets, the partitions of which are constructed of two layers of teak between which are sandwiched

Fig. 1.—Ground Floor Plan.

Fig. 2.—First Floor Plan.

sheets of lead; the windows are of lead-glass, and the actions of the rays are thus confined to the interior of the cabinets. Each cabinet is supplied with a "Roentgen" bulb, which can be manipulated from the outside, obviating any necessity for the operator being exposed to the action of the rays, and further allowing of his attendance on more than one patient at the same time. There is an automatic arrangement for cutting off the current at any given time, managed by a clockwork attach-

FIG. 3.—DIRECTOR'S CONSULTING ROOM.

FIG. 4.—GENERAL THERAPEUTIC ROOM. Showing X-ray Cabinets at the end, Diathermy, High-frequency, Mercury Vapour Lamp, Universal Therapeutic Board, Four-cell Bath, Camera, etc.

ment; and there is a device by which the opening of the door switches off the electric supply.

Careful arrangements have been made for ventilation. The windows of the building are exceptionally large, and an exhaust fan, fitted in the roof, permits of the circulation of fresh air throughout the building. The building is heated by hot-water radiators supplied from boilers situated in the basement. The ground floor and passages are of terrazzo laid in squares between marble strips; this method has been found to limit the extension of cracks to which this flooring is liable in new buildings when settlement occurs.

The staircase is built of stone, and the walls in the hall and passages are tiled to a height of about four feet; above this they are distempered. All the woodwork is of hard teak. The cost of the building was £3,000.

The staff of the department comprises the director, two clinical assistants, and the physicist, all of whom are honorary officials; there are also a sister in charge, and the dispenser of the hospital, who has made a special study of electrical work, acts as a lay assistant; a boy is employed to assist in the physical laboratory. The opening of the new wards will, of course, necessitate an increase in the nursing staff. At present those patients who require indoor treatment reside in the hospital wards and only attend the department for treatment.

The treatment is chiefly by $x$-rays and radium-therapy, though other methods of electrical treatment are used to aid these. The former method is divided into (1) prophylactic, (2) palliative, (3) curative. The first is the administration of $x$-rays at regular intervals after operation with the object of preventing recurrence. It has been found that patients so treated improve in general health, and in some cases recover freer movement of the limbs more rapidly. The feature of palliative treatment is the relief of pain in a large number of cases, the diminution in the size of the tumour in a small percentage, and, in some instances, the rendering of an inoperable condition operable, and the bringing about of an improvement in general health. Curative measures apply to but a small group of cases, but in several instances superficial lesions have been cleared up, for at least a time. This particularly applies to early cases of rodent ulcer and superficial skin cancers.

The duration of exposure to the $x$-ray treatment has now been greatly increased through the use of a more penetrating ray through filters; where an exposure of ten minutes was formerly made, half an hour can now be given.

A large number of cases are treated by radium alone or in combination with other methods, in several cases showing marked improvement. Several patients treated in former years are still attending and remain well.

The radiographic work is of immense importance for purposes of diagnosis. It frequently guides the surgeon as to the extent of his operation, and in some cases enables a definite prognosis to be arrived at. Examination by means of the bismuth meal is largely employed, and in many cases has helped in the definite diagnosis of cancer of the stomach, œsophagus, or intestine.

An interesting branch of the work is that of the collection of radium emanations. By means of a mercury pump, designed by the physicist, emanations are extracted from 65 milligrammes of radium bromide in solution which is permanently attached to the pump. These emanations, which are equally efficacious as radium itself, are frequently used in the course of treatment, but they have the disadvantage of rapidly losing their strength. The activity of the emanation persists long enough to enable exposures of three or four days being given: the advantage is that the patient may be allowed to leave the hospital with the emanation tube in position, which of course could not be allowed in the case of radium.

Fig. 5.—One of the Radium Cubicles.

Attendances, Old Patients, during the Year 1913.

| | |
|---|---|
| 596 | for $x$-rays, $x$-rays and diathermy, and $x$-rays and electricity. |
| 466 | for $x$-rays and radium and $x$-rays and radium emanation. |
| 11 | for electricity. |
| 257 | for radium. |
| 1,430 | |

Attendances, New Patients, during the Year 1913.

| | |
|---|---|
| 959 | for $x$-rays, $x$-rays and diathermy, and electricity. |
| 348 | for $x$-rays and radium and radium emanations. |
| 455 | for radium, radium emanations, and mercury lamp. |
| 233 | for electricity. |
| 287 | radiographed. |
| 2,282 | |

Number of radiographs taken during the year, 1,172.

[The illustrations are reproduced by kind permission of the Editor of *Archives of the Roentgen Ray*.]

## Figures and Tables

**Fig. 1. f1:**
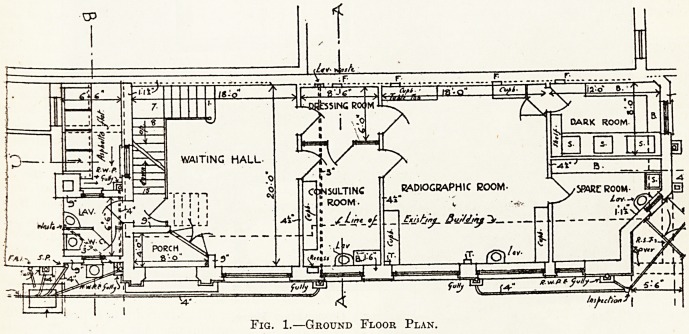


**Fig. 2. f2:**
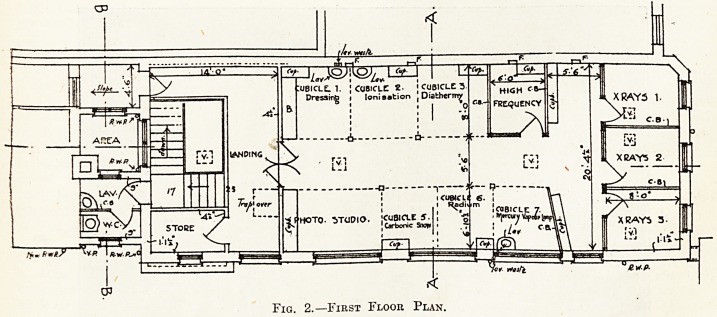


**Fig. 3. f3:**
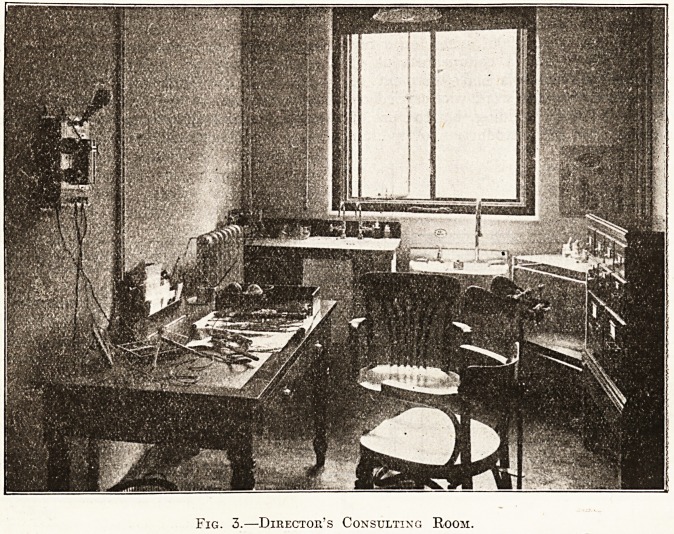


**Fig. 4. f4:**
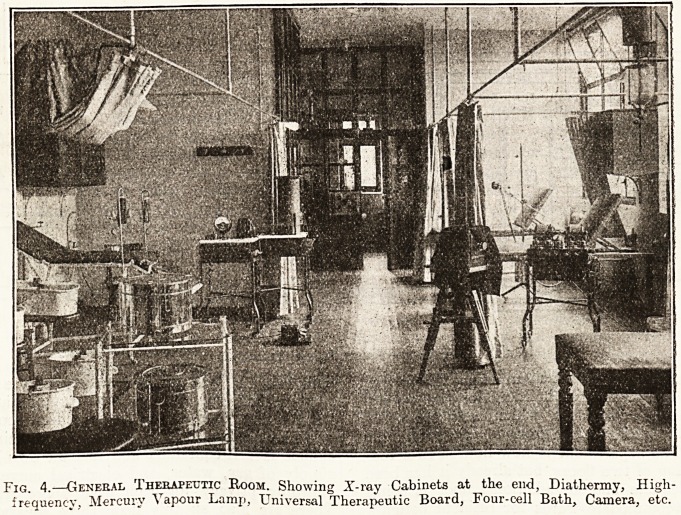


**Fig. 5. f5:**